# Supplementary material for: Palm Oil-Rich Diet Affects Murine Liver Proteome and S-Palmitoylome
Source: Int J Mol Sci. 2021 Dec 3;22(23):13094. doi: 10.3390/ijms222313094 (PMC8657750; doi:10.3390/ijms222313094)
Supplement: Supplementary file 1 [file ijms-22-13094-s001.zip › Supplemental Materials and Methods_Warsaw.pdf]

Palm oil-rich diet affects murine liver proteome and *S*-palmitoylome

Supplemental Materials and Methods

Content:

1. Composition of RD and HPD diets according to ssniffSpezialdiaten GmbH
2. Primers used for RT-qPCR analyses

1. Composition of RD and HPD diets according to ssniffSpezialdiaten GmbH (Soest, Germany)

Ingredient (g/100 g)	RD	HPD
Casein	20.7	25.6
Corn starch, pre-gelatinized	40.6	16.0
Maltodextrin, 10DE	17.21	17.26
Sucrose	5.0	5.0
Cellulose powder	5.0	5.5
L-Cysteine	0.25	0.30
Vitamin premixture	1.0	1.0
Mineral and trace element premix	6.0	6.0
Choline Cl (50%)	0.2	0.2
Butylated hydroxytoluene	0.01	0.01
Food dye (yellow/red)	0.03	0.03
Palm oil	-	19.1
Soybean oil	4.0	4.0
**Energy (Atwater)(MJ/kg)**		
total	15.2	19.2
kcal% protein	20	20
kcal% fat	10	45
kcal% carbohydrates	70	35

RD and HPD diets differed in palm oil content delivering 10 and 45 kcal% from fat, respectively. They both delivered 20 kcal% from proteins and contained the same amounts of sucrose, i.e., 5% by weight. The energy content (reaching 15.2 MJ/kg in RD and 19.2 kJ/kg in HPD) was adjusted by the different content of complex carbohydrates from pre-gelatinized corn starch. Thus, RD diet delivered 70 kcal% from carbohydrates, while HPD – 35 kcal%.

2. Primers used for RT-qPCR analyses

	sequence 5'→3'
**mHPRT_F**	CAGTCCCAGCGTCGTGA
**mHPRT_R**	GCCTCCCATCTCCTTCAT
**mTBP_F**	CAGAACAACAGCCTTCCACC
**mTBP_R**	GAGTAAGTCCTGTGCCGTAAG
**mTLR4_F**	GCTTACACCACCTCTCAAACT
**mTLR4_R**	GTCTCCACAGCCACCAGATT
**mCD14_F**	CCAAGCACACTCGCTCAACT
**mCD14_R**	ATCAGTTCCTCTCTCGCCCAA
**mCD36_F**	CTAAATGAGACTGGGACCA
**mCD36_R**	ACATCACCACTCCAATCC
**mTNFα_F**	TGTCTCAGCCTCTTCTCATTC
**mTNFα_R**	TGAGGGTCTGGGCCATAGAAC
**mRantes_F**	GCTCCAATCTTGCAGTCGTGT
**mRantes_R**	CCATTTTCCCAGGACCGAGT
**mIL-1β_F**	CTTCAGGCAGGCAGTATCA
**mIL-1β_R**	AGGATGGGCTCTTCTTCAAA
**mMIP-2_F**	CCACCAACCACCAGGCTACAGGGGC
**mMIP-2_R**	AGGCTCCTCCTTTCCAGGTCAGTTAG
**mSMS1_F**	TAGTTGGCACGCTGTACCTG
**mSMS1_R**	TGTTGTGCGAGCCTGTGATG
**mSMS2_F**	ACCCAAGACCTTATCCAACG
**mSMS2_R**	AGGATGAGGTTGAAGAGCG
